# Leveraging psychedelic neuroscience to boost human creativity using artificial intelligence

**DOI:** 10.3389/frai.2025.1589086

**Published:** 2025-06-04

**Authors:** Brian M. Ross

**Affiliations:** Division of Medical Sciences, NOSM University, Thunder Bay, ON, Canada

**Keywords:** artificial intelligence, creativity, divergent thinking, implicit learning, latent inhibition, psychedelic

## Abstract

Psychedelics, such as LSD and psilocybin, disrupt entrenched cognitive patterns by facilitating novel insights and new associations. This paper considers how AI can potentially mimic these psychedelic-induced cognitive disruptions to augment and enhance human creativity. Psychedelics likely enhance creativity by altering brain function, notably the activity of the Default Mode Network, which leads to changes in cognition. Psychologically, they may reduce latent inhibition, increase divergent thinking, and promote implicit learning. Similarly, AI systems can replicate these creative enhancements by introducing novel associations, reframing familiar information, and facilitating unconscious cognitive shifts. The risks associated with AI use are also compared to psychedelics, including dependency, ethical concerns, and homogenization of outputs due to bias. Integrating the cognitive mechanisms activated by psychedelics into AI design provides promising pathways for creativity enhancement. Carefully designed AI could act as a cognitive catalyst, fostering innovative thought processes and adaptive problem-solving while addressing identified ethical and practical concerns.

## 1 Introduction

Humanity is confronted by what often seems like intractable systemic crises in health, the environment, and the global economy. We appear cognitively stuck, navigating the world through what Timothy Leary described as “reality tunnels”: subjective frameworks built from habituated perception, cultural conditioning, and neurobiological constraints. According to Leary, escape from these tunnels requires a radical reimagining of the problems we face (Higgs, 2024). A similar idea appears in Deleuze and Guattari’s *A Thousand Plateaus*, where creativity is said to emerge through “lines of flight,” departures from structured thought that break through rigid systems of meaning. These processes of “deterritorialization” disrupt familiar cognitive patterns, opening space for new configurations to emerge (Deleuze and Guattari, 1980). Leary suggested that psychedelics offer one means to make transformative changes of thought, in that they allow for a temporary suspension of entrenched cognitive habits, enabling users to reinterpret the ordinary and uncover previously hidden relationships between concepts and ideas (Higgs, 2024). Generative AI, while mechanistically different, may offer analogous ruptures in meaning-making by loosening conventional semantic constraints. Though their ontologies differ, both psychedelics and AI may enhance transformative creative potential by introducing productive cognitive destabilization (Gruetzemacher and Whittlestone, 2020). This conceptual overlap is intriguing, but how can AI be meaningfully integrated with our existing human capacities for change and creativity? Put another way, can AI work *with* human psychology, rather than simply around it?

In this paper, I argue that psychedelic substances offer both a conceptual and neurocognitive model for designing AI systems that augment human creativity. Specifically, I explore how the cognitive and neural mechanisms influenced by psychedelics, such as reduced latent inhibition, increased divergent thinking, and enhanced implicit learning, can inspire approaches to AI aimed at supporting novel insight generation. To do so I will review the neuroscience and psychology of psychedelics before outlining the psychological mechanisms underpinning creative thought. I will then suggest how AI systems may replicate or enhance these mechanisms, and how AI adapted to individual personality traits may optimize the impact of AI on a person’s creativity. Finally, I will discusses the potential risks of this approach, including bias and dependency.

## 2 Psychedelic psychology and neuroscience

Psychedelic drugs are psychoactive compounds that dissolve rigid cognitive structures, giving rise to altered states of consciousness marked by sensory distortions, visual imagery, and intensified meaning-making (Kelmendi et al., 2022; Nichols, 2016; Dittrich, 1998). Historically used in spiritual and ritual contexts, they have since been studied for their effects on perception, cognition, and the sense of self, as a means to explore human consciousness (Mijares, 2015). LSD, first synthesized in 1938, is a classical psychedelic that acts on serotonin receptors, much like psilocybin (found in “magic mushrooms”) and DMT (present in Ayahuasca, traditionally used in Amazonian ceremonies) (Nichols, 2016; Labate and MacRae, 2016). Other compounds with psychedelic-like properties include MDMA, which enhances mood and empathy via serotonin, dopamine, and norepinephrine, and that of ketamine, an anesthetic with rapid antidepressant effects thought to act through glutamatergic modulation (Bowdle et al., 1998; De la Torre et al., 2000).

A key feature of psychedelics is their ability to disrupt entrenched neural patterns, fostering novel thinking and opening potential pathways for treating mental illness (Nutt and Carhart-Harris, 2021). These substances appear to induce a more entropic state of consciousness, loosening fixed cognitive frameworks and promoting divergent, nonlinear thought (Carhart-Harris et al., 2014). Indeed, despite their pharmacological differences, most psychedelics share a common neurological mechanism: disruption of the Default Mode Network (DMN), a brain system associated with self-referential thought, abstract reflection, and the construction of one’s inner world (Gattuso et al., 2023). The DMN contrasts with the Central Executive Network (CEN), which governs goal-directed behavior in the outside world, and the Salience Network, which toggles attention between the DMN and CEN depending on current needs (Goulden et al., 2014). Psychedelics disrupt, and generally reduce, activity in the DMN, while enhancing communication between otherwise segregated brain regions. This leads to diminished self-referential thinking, increased feelings of unity, and a greater capacity for creative insight (Askenasy and Lehmann, 2013). DMN disruption is central to the therapeutic use of psychedelics, especially in the treatment of conditions like depression, anxiety, and PTSD using substances such as psilocybin and MDMA (Reiff et al., 2020). For example, individuals with chronic anxiety often exhibit hyperactive DMN activity, reinforcing repetitive negative thought loops. A carefully guided psychedelic experience can temporarily weaken these patterns, offering a window into new cognitive perspectives and more adaptive mental frameworks (Reiff et al., 2020).

Importantly, the disruption of the DMN may underpin recent evidence that psychedelics enhance creativity (Mason et al., 2021). Several studies suggest they facilitate novel associations by relaxing cognitive constraints and encouraging a more fluid, exploratory mindset (Carhart-Harris et al., 2014; Gandy et al., 2022; Girn et al., 2020; Kuypers et al., 2016). However, these effects are not uniform. Creativity outcomes vary based on factors such as personality traits, mindset, intention, and environmental context, commonly referred to as “set and setting” (Johnstad, 2021; Hartogsohn, 2016). While some research suggests that psychedelics increase divergent thinking and insight, others report negligible effects or emphasize the importance of deliberate context and guidance (Mason et al., 2021). Rather than weakening the case for psychedelics as creativity enhancers, this variability highlights a deeper insight: creativity is emergent, context-sensitive, and shaped by both internal and external conditions. The importance of the interplay between context, personality and cognitive change is key when considering how human creativity can be augmented using AI as it suggests that any such system should be not only disruptive, but also adaptive to the person and their current context.

In summary, psychedelic substances alter brain function by disrupting entrenched neural patterns, particularly through reduced activity in the DMN, a system associated with self-referential thinking and habitual cognition. This disruption increases connectivity between brain regions, loosens cognitive constraints, and promotes a more flexible state of consciousness characterized by divergent thinking and the generation of novel associations. Such changes which may enhance the creativity of users in ways dependent on various contextual factors.

## 3 Psychological foundations of creativity

Human creativity spans a continuum from routine problem-solving to highly original and transformative thinking, influenced by cognitive flexibility, personality traits, and domain-specific expertise. Research indicates that individuals with higher cognitive flexibility are better equipped to generate innovative ideas and adapt to new situations, underscoring the importance of these factors in creative performance (Gandy et al., 2022; Sung and Choi, 2009). Creativity varies widely among individuals and is closely associated with openness to experience, a “Big Five” personality trait linked to curiosity, abstract thinking, and a preference for novelty (Sung and Choi, 2009). Those high in this trait tend to exhibit distinct cognitive characteristics, which are measurable across three domains relevant to creativity: latent inhibition, divergent thinking, and implicit learning.

Latent inhibition refers to the brain’s ability to filter out stimuli previously judged as irrelevant, helping to prevent sensory overload and maintain focus on what is deemed important. While useful for everyday functioning, high latent inhibition can constrain creative thought by limiting access to alternative associations. In contrast, low latent inhibition enables individuals to maintain a broader stream of sensory and cognitive information in conscious awareness, creating fertile ground for the generation of novel combinations of ideas and unexpected insights (Carson et al., 2003; Lubow and Weiner, 2010).

Creativity also depends on a dynamic interplay between divergent and convergent thinking. Divergent thinking is the capacity to generate multiple, varied, and original ideas, and is characterized by cognitive flexibility and associative leaps (Kenworthy et al., 2021). Convergent thinking, by contrast, involves narrowing down options to identify the most effective or appropriate solution, emphasizing logic, structure, and evaluative reasoning (Razumnikova, 2020). While both processes are essential to problem-solving, divergent thinking is particularly critical for producing creative breakthroughs, whereas convergent thinking is related to selection and implementation including the consideration of matters of related to feasibility, suitability, and ethics.

Implicit learning, the third key process, refers to the unconscious absorption of patterns and structures through experience (Kaufman et al., 2010). It supports creativity by allowing individuals to internalize complex relationships without deliberate effort, later applying this knowledge in new contexts without being able to explain precisely how. This process enhances associative thinking, enabling disparate concepts to combine into fresh insights (Frensch and Rünger, 2003). It also and contributes to intuitive problem-solving, where solutions are recognized before they are fully understood or verbalized. Implicit learning also appears to reduce cognitive rigidity, fostering originality by allowing people to bypass conventional frameworks (Oleynick et al., 2017). Reinforcing the importance of these processes is emerging research suggesting that psychedelics, particularly serotonergic compounds like LSD, may amplify a person’s creativity by temporarily reducing latent inhibition, enhancing associative processing, and promoting changes in brain connectivity that facilitate both divergent thinking and implicit learning (Kuypers et al., 2016; Hitchcock et al., 1997; Costa, 2020; McGovern et al., 2023).

How then can psychedelic-induced cognitive change provide a conceptual foundation for exploring AI-assisted creativity? Just as psychedelics disrupt entrenched cognitive frameworks to allow for new ways of thinking, AI can serve a similar function by identifying novel patterns and associations that can help their human collaborators identify alternative solutions thereby augmenting the creative process. This includes aiming to reduce our tendency to filter out information (latent inhibition), assist with idea generation although maybe not selection (divergent vs. convergent thinking), and prime our ability to discern novel linkages and structures to allow users to internalize complex relationships and generate creative insights without conscious effort (implicit learning). However, AI’s potential may extend beyond mere augmentation as it can fundamentally change how we think by altering cognitive structures in a manner analogous to psychedelics. Such human cognitive transformation by AI has been argued for previously (Carter and Nielsen, 2017) and I suggest that this is most likely to occur if AI is designed in such a way so that it targets the brain processes underlying creativity. In the next section I will explore each of these mechanisms in the context of AI systems.

## 4 Artificial intelligence as psychedelic-mimetics

### 4.1 AI and latent inhibition

Our tendency to filter out information that we have previously considered, i.e., latent inhibition, may be amenable to being overcome using AI systems as well as by psychedelics. Indeed, AI can present unfiltered information, devoid of the user’s pre-existing cognitive biases that limit what persons either recognize as important or seek out at all. In addition, AI-generated content, especially in art, music, and text-based creativity tools, can simulate this effect by producing a constant stream of novel inputs that disrupt habitual thought patterns. For example, Jukebox AI generates unexpected music compositions and brings musical forms into consciousness that a person may have discounted without mindful awareness (Dhariwal et al., 2020). Moreover, consider a furniture designer who normally filters out ideas based on conventional chair designs (high latent inhibition). To help her think differently, she could use an AI system that deliberately disrupts this process by suggesting new designs based on unconventional or seemingly unrelated concepts, such as “cloud,” “bicycle” and “origami.” This bypassing of the mind’s usual filtering processes can therefore allow her to conceptualize novel designs that she would otherwise have overlooked. That being said, a person can still ignore such AI generated associations that they discount as outlandish, and it is here that AI can reframe concepts in multiple ways, encouraging users to consider alternative interpretations rather than relying on fixed mental models. AI also has the potential to prime ideas generation by recontextualizing data in real-time. For example, in AI-assisted brainstorming, models like GPT-4 can generate different re-phrasings of a problem which may be able to bypass latent inhibition, in effect bringing more information into conscious awareness than would otherwise occur (Shaer et al., 2024).

### 4.2 AI and divergent thinking

The potential to detect novel connections between informational elements (facilitated by reduced latent inhibition) is also something that AI-driven algorithms can augment. AI systems excel at detecting patterns that may escape human attention, due to their capacity to analyze large high-dimensionality datasets using probabilistic inference. While they are not subject to intrinsic cognitive biases such as anchoring or attentional narrowing, they may still inherit and amplify biases present in their training data or in human-guided learning. This distinction is important: AI bias is not psychological but structural, and potentially correctable through transparency, oversight, and algorithmic design (Binns, 2018). This is seen with DeepDream image recognition which produces psychedelic-like visual artifacts by amplifying hidden patterns, making users see configurations they may have otherwise ignored due to bias (Al-Khazraji et al., 2023). Indeed, tools like DeepDream and generative language models can produce surprising associations that simulate aspects of divergent thinking (Al-Khazraji et al., 2023). Large language models can also combine unrelated concepts in ways that simulate creative leaps, particularly as AI models have no intrinsic need for social constraints or exhibit human-like cognitive biases, allowing them to suggest unusual but potentially insightful ideas (Chen and Ding, 2023; Grilli and Pedota, 2024; Hubert et al., 2024). For example, creative writing tools using AI often suggest novel juxtapositions of language, encouraging users to pursue ideas they may not have consciously generated (Chen and Ding, 2023; Kobierski, 2023). Similarly, architectural tools like Midjourney offer visual reimagining based on textual input, exposing designers to a broader conceptual space (Jaruga-Rozdolska, 2022). However, while these systems do not engage in human-like creativity *per se*, they can act as catalysts for human divergent thinking by introducing unexpected prompts, combinations, or perspectives. While our understanding of the interplay between AI and human process is still in the early stages, these examples illustrate how AI can create environments that support or stimulate divergent thinking, especially when integrated thoughtfully into human workflows.

In future systems, divergent thinking could be further enhanced by implementing dynamic “conceptual mutation” layers, algorithms that intentionally inject low-probability, cross-domain associations based on user input and context (Meincke et al., 2024). Indeed, designing AI systems with built-in capacities for controlled disruption may foster greater generativity and novelty, paralleling how DMN disruption by psychedelics fosters novel thinking. These layers could be modulated in real time based on the interaction with the user, mimicking the “associative looseness” seen under psychedelics. Moreover, incorporating emotional-responses, such as surprise, pleasure, or esthetic dissonance, may help prioritize outputs that more closely mirror the psychological richness of psychedelic insights (Kuypers et al., 2016; Schmidhuber, 2010; Maher et al., 2013).

### 4.3 AI and implicit learning

To recap, implicit learning refers to unconscious knowledge acquisition through repeated exposure and pattern detection without explicit awareness of what is being learned. AI can model implicit learning through mechanisms like reinforcement learning, long-term user modeling, and personalized content curation. These systems adapt over time without requiring explicit instruction, similar to how humans internalize patterns unconsciously. AI models already can adjust to a user’s interest and cognitive styles such as seen with the Replika chatbot (Babu and Prasad, 2024). In doing so an AI can act as an adaptive mentor to guide learning in subtle and unconscious ways. The directing and enhancement of implicit learning is seen in various ways in AI applications. For example, AI models like Replika can be used in a mentoring role which operate implicitly as well as explicitly (Babu and Prasad, 2024). AI recommendation systems in social media platforms shape behavior and preferences through unnoticed pattern reinforcement. This can be used to enhance creative thought by incorporating novel elements into user’s feeds, to escape the “reality tunnels,” rather than acting as an echo chamber to keep persons within them (Cinelli et al., 2021). Implicit learning is also seen in AI language models like ChatGPT which have been employed as interactive tutors, providing conversational practice that aids users in absorbing linguistic and narrative patterns implicitly. For example, learners practicing Japanese with ChatGPT reported improved confidence and understanding through regular, informal interactions (Rifai et al., 2024). Moreover, such systems allow novel story ideas to surface that conform to genre rules and display coherence without formal instruction (Gervás, 2009). AI systems can also learn and adapt to cultural values and ethical norms (Zhang et al., 2024), providing a learning and mentoring tool for those originating from different cultural groups (Klimova and Chen, 2024).

In addition to aiding in learning about the structural rules guiding creativity, AI may also have the potential for increasing creativity itself via implicit learning. Future AI models could be designed that gradually introduce novel patterns at the edge of user familiarity, enhancing creativity without overwhelming the user. This mirrors how implicit learning occurs in humans, where repeated exposure reshapes understanding without conscious awareness (Cleeremans and Jiménez, 2013). Adaptive learning systems, such as those used in personalized education and media platforms, already approximate this principle by considering long-term user behavior to refine outputs (Brusilovsky and Millán, 2007). Extending these approaches into creative domains, such as interactive storytelling or metaphor generation, could allow AI to support novel conceptual frameworks in subtle, immersive ways. For example, an AI-based music streaming service may offer a composer the option of subtly exposing them to musical structures, rhythms, and harmonies outside their typical listening preferences. Over time, without consciously noticing it (implicit learning), they may mentally internalize these novel musical patterns which they can then integrate into their own compositions, enhancing creativity without explicit awareness of how this has occurred. This is also seen in combined AI/virtual reality-based simulations that seek to teach communication and cultural competency skills through role playing (Beavers et al., 2024).

Implicit learning is, however, something of a double-edged sword as it can be used to influence behavior without the user being aware that this is occurring. There is therefore a need to include strong ethical safeguards, that transparently inform users about the methods and aims of the system. We can also learn from our experiences with psychedelics as these substances can lead to users developing untrue beliefs through both implicit and explicit processes. This has resulted in the need to develop codes of conduct which inform and enforce ethical behavior of those conducting either psychedelic-based ceremonies or using them therapeutically such that they do not abuse their position for personal gain or to cause harm, something that may be usefully mimicked in AI-system design (McGovern et al., 2023; Kochevar, 2024; Rochester et al., 2022).

In summary, I have argued that some of the cognitive mechanisms influenced by psychedelics have their parallels within AI systems:

- Reducing latent inhibition by bypassing user biases and introducing novel, unfiltered inputs, facilitating unexpected associations
- Enhancing divergent thinking via cross-domain recombination to produce novel insights
- Strengthening implicit learning by means of adaptive, long-term interactions during which AI systems personalize outputs and reinforce unconscious pattern recognition

These suggest three design principles for creativity-enhancing AI:

- Introduce controlled noise or novelty to disrupt mental filters
- Generate cross-domain outputs in a manner which has affective salience
- Embed adaptive feedback loops to foster cumulative, intuitive insight

These principles, inspired by psychedelic neuroscience, may serve as useful heuristics in the development of AI tools that augment human creativity.

## 5 Interaction between personality and AI

I have suggested that AI can enhance creativity in humans by mimicking or enhancing the underlying core psychological and neurological processes. Creativity is not distributed equally in the human population. This has many life impacts including those connected to equality, career choice and income, and adaptive ability (including the new environment being fostered by technological advances in AI) (Ternavska et al., 2024; World Economic Forum, 2016). I suggest that AI may be able to act as an equalizer of sorts, allowing those with limited creative abilities to exceed their baseline capacity.

That being said, whether or not such persons wish to do so is also entwined with their trait personality. For example, those with low trait open to experience may be hesitant to use such technology due to having lesser comfort with the act of mental exploration. Again we can draw on experiences with psychedelics which suggest that those already amenable to risk taking and gaining new insights and perspectives are more likely to use these substances (Johnstad, 2021). It is therefore also possible that both psychedelics and AI will widen the personality-related creative gaps between people rather than close them, mirroring the risk that AI will expand demographic inequities (Cachat-Rosset and Klarsfeld, 2023). Being aware of this potential downside does allow designers to incorporate mitigation strategies such as by introducing new ideas in a manner that is adapted to the user’s personality. However, as we saw in Section 2, the use of psychedelics has suggested that contextual set and setting are also important modulators of creativity enhancement. As such, any AI system may have to account for both context and personality, modulating outputs based on user goals, psychological states, trait personality, and cognitive styles. This offers an opportunity not just to replicate but to refine the conditions that enable creative breakthroughs. Consider, for instance, a person who scores low on openness to experience but high on trait conscientiousness. Such individuals prefer structure, order, and predictability though this can change with contextual familiarity and comfort. A creativity-enhancing AI agent could tailor its interactions by initially offering ideas that are only mildly divergent from the user’s existing conceptualization of the matter they are working on, perhaps by adjusting parameters like semantic distance, novelty intensity, associative breadth, emotional tone, or ambiguity level that are tailored to user comfort. Indeed, these systems could also include feedback mechanisms, allowing users to signal wellbeing, or feeling overwhelmed or bored, so that the system can continuously recalibrate. This could be done through explicit user feedback, or by monitoring physiological markers such as facial expressions linked to emotions such as anxiety or pleasure (Tao et al., 2024). Over time, and with positive reinforcement, the system could gradually expand the novelty range, creating a scaffolded experience of creative exploration that respects personality boundaries while still encouraging growth.

Ultimately, building AI tools that are personality-sensitive may not only enhance individual creativity but also make such tools accessible to a wider range of users who might otherwise avoid such applications entirely. To support this AI systems need to include a psychometric layer, based on prior user behavior, language patterns, explicit personality questionnaires, or automated response testing, allowing them to infer traits such as openness, conscientiousness, neuroticism, or risk tolerance, augmented by ongoing real-time feedback. Recent advances in affective computing and personality-aware recommendation engines support the feasibility of such adaptive systems (Mairesse et al., 2007). For instance, emotion-sensitive AI chatbots like Woebot and Sonar already adjust their tone and conversational strategies in real-time to facilitate mental health support to those with different affective needs (Suharwardy et al., 2023; Sonar Mental Health, 2024). A similar architecture could be applied to creativity augmentation, where idea generation is modulated by the user’s personality. This approach mirrors the “zone of proximal development” in educational theory, which suggests that learning best occurs when it is pitched just beyond the learner’s current capacity and when scaffolded by appropriate support (Vygotsky, 1978).

Currently, there is very little information regarding how a putative creativity-enhancing AI will change a person’s existing psychology and/or brain function in addition to augmenting it. There is a need for research using psychological testing or functional neuroimaging which directly investigates how AI use changes us at the psychological and/or neurological level, including that of personality, be these traits, values, or the stories we tell about ourselves. Such work is a necessary precursor for using brain-machine interfaces that directly connects AI to human neuronal architecture (Asgher et al., 2023; Eldawlatly, 2024). For example, future studies could experimentally compare combinations of two key independent variables: (i) AI creativity range (low, moderate and high novelty) and (ii) user openness level (high vs. low, as measured by a Big Five inventory), while measuring user engagement, self-reported and automated measures of comfort, and creative output metrics. This would allow researchers to examine whether tailoring creativity support to personality improves outcomes, and whether it reduces, preserves, or widens baseline creative disparities.

## 6 Cautions

Psychedelics, with the exception of ketamine (Morgan et al., 2012), have a low potential for addiction. Although some artists use psychedelics regularly as a means of enhancing creativity (Cortes, 2018), this pattern of use typically does not become problematic. Indeed, most classical psychedelics have low addictive potential, even with repeated use, likely due to their intense and often challenging psychological effects which naturally limit excessive or compulsive use (Bouso et al., 2018; Dittrich, 1998). In contrast, AI is easily accessible and may foster dependency. Studies suggest overreliance on AI can weaken critical thinking and problem-solving skills, as users increasingly outsource cognitive effort to algorithms (Jia and Tu, 2024; Zhang et al., 2024; Epstein et al., 2020). To prevent this, AI literacy training should emphasize workflows where AI provides suggestions, but humans critically evaluate and finalize decisions. That is, while AI may facilitate divergent thinking, humans must maintain control of the convergent endpoints.

Another concern is homogenization of creative output. AI generates ideas based on existing data, potentially reinforcing biases and producing derivative rather than truly original work (Anderson et al., 2024). Indeed, AI models trained on AI-generated content can quickly degrade into producing nonsensical outputs (Shumailov et al., 2024). While human creativity also builds on prior knowledge, the most creative humans currently outperform AI in generating original ideas (Koivisto and Grassini, 2023). This may be due to the depth and variety of human experience, which surpasses the internet-derived training data used for AI models (Boden, 2018). Moreover, AI can inherit historical and cultural biases from its training data, potentially marginalizing underrepresented voices (Buolamwini and Gebru, 2018; Noble, 2018). To address this, AI should be trained on data deriving from diverse human experiences, and human oversight in AI development should include evaluators from varied backgrounds to ensure inclusivity (Bender et al., 2021).

## 7 Closing comments

Understanding how psychedelics alter cognition may be able to inform the design of AI applications that enhance creativity and problem-solving without pharmacological intervention. As this paper has outlined, psychedelics can reduce latent inhibition, promote divergent thinking, and enhance implicit learning in context-dependent ways, mechanisms that can inspire the development of AI systems supporting more flexible, exploratory, and intuitive cognition. Future research should empirically test how these mechanisms can be translated into algorithmic form. Experimental designs could include user studies that manipulate novelty levels or idea-space breadth in AI interactions, measuring their impact on creative output, particularly across personality dimensions such as openness to experience. Neuroimaging studies might also explore how interacting with creativity-enhancing AI affects neurological and psychological functioning, such as those associated with the DMN.

A particularly promising direction involves adapting AI systems to individual personality traits. Just as psychedelics may differentially impact users depending on psychological predisposition, AI tools could modulate how they introduce novelty, abstraction, and ambiguity based on inferred personality profiles. This could make creativity-enhancing AI more accessible to people who might not otherwise seek or benefit from traditional tools with implications for equity and inclusion. Practical applications include education systems that help students loosen rigid learning patterns, design tools that function as intelligent creative prompts, and therapeutic settings where perspective-shifting AI could act as a scaffold for change. Ultimately, by integrating insights from psychedelic neuroscience with adaptive, user-sensitive AI design, we may move toward a new class of technologies, ones that helps us both understand and expand the creative potential of the human mind (Gobet and Sala, 2019).

## Data Availability

The original contributions presented in the study are included in the article/supplementary material, further inquiries can be directed to the corresponding author.
